# The marginal gaps of sequentially milled lithium disilicate crowns using two different milling units

**DOI:** 10.1111/adj.12909

**Published:** 2022-03-28

**Authors:** K Tan, J Dudley

**Affiliations:** ^1^ Adelaide Dental School The University of Adelaide Adelaide SA Australia

**Keywords:** Bur wear, crown, lithium disilicate, marginal gap, milling unit

## Abstract

**Background:**

The purpose of this study was to compare the marginal gaps of sequentially milled lithium disilicate (LDS) crowns using two different milling units.

**Methods:**

One lower left first molar typodont tooth prepared for an LDS crown by an undergraduate student in a simulation clinic was selected. The crown preparation was scanned by a TRIOS 3 scanner and twelve LDS crowns milled by an E4D (E4DM) and a Sirona inLab MC X5 (MCX5) milling unit using identical settings. The crowns were seated onto the original crown preparation and three vertical marginal gap measurements were taken at four locations (mid‐buccal, mid‐lingual, mid‐mesial and mid‐distal) using a stereomicroscope. The mean marginal gap (MMG) was calculated for each individual tooth surface and each crown.

**Results:**

The MMG for the E4DM (100.40 μm) was not significantly different to the MCX5 (101.08 μm) milling unit (*P* = 0.8809). In both units, there was a statistically significant trend of increasing MMG with sequentially milled crowns using the same burs (E4DM *P* = 0.0133; MCX5 *P* = 0.0240).

**Conclusions:**

The E4DM and MCX5 milling units produced LDS crowns with similar MMG’s and within a clinically acceptable range but with a trend of increasing MMG when analysed sequentially. © 2022 Australian Dental Association

**Abbreviations and acronyms**
CAD: computer‐aided design
CAD/CAM: computer‐aided design/computer‐aided manufacturing
LDS: lithium disilicate
MMG: mean marginal gap
STL: standard tessellation language

## INTRODUCTION

The advent of computer‐aided design/computer‐aided manufacturing (CAD/CAM) technology in the 1980s expanded the options and materials available for constructing all‐ceramic crowns. More recently, the range of milling units has grown exponentially with manufacturer claims of improved efficiency, accuracy and versatility leading to great competition in the industry. While chairside milling units are convenient, laboratory‐based milling units are generally designed to handle greater production requirements.^1^ The ability to scan a crown preparation and mill a crown chairside has clear advantages including the elimination of the need for traditional impressions, reduced number of appointments and clinical time, reduced or no need for temporisation, increased patient convenience, utilisation of digital technologies and delivery of a same day crown. However, the comparison of the accuracy of the chairside technique with generally larger laboratory‐based milling units has been limited.

IPS e.max CAD (Ivoclar Vivadent, Schaan, Liechtenstein) is a pre‐sintered millable lithium disilicate (LDS) ceramic material first introduced in 2006.^2^ IPS e.max has excellent physical properties and a wide range of aesthetic options making it a popular choice amongst clinicians. The material is milled in a partially crystallised ‘blue state’ comprising metasilicate (Li_2_SiO_3_) crystals which are later converted to disilicate (2SiO_2_‐Li_2_O) crystals during the heat crystallisation process.^3^

Of the many factors that contribute to the clinical success of crowns, the marginal fit of the crown to the prepared tooth surface is considered the key determinant.^4^, ^5^ Accurate marginal fit reduces the risk of hypersensitivity due to microleakage^6^ and reduces local plaque accumulation at the crown‐tooth interface. A failure to ensure accurate marginal fit can result in the development of periodontal disease and potentially dental caries which might lead to pulpitis.^4^, ^7^ There has been a wide range of ceramic material marginal gaps reported in the literature ranging from 7.5 to 206.3 μm largely due to the heterogeneity of studies.^8^ In particular, there have been variations in the definition of marginal gap, different methods employed to determine the size of the marginal gap involving invasive and non‐invasive techniques, marginal gaps measured both pre‐ and post‐cementation, and the use of ceramic systems that involve different construction techniques that affect the accuracy of fit.^8^ However, a widely accepted maximum clinical marginal gap is 120 μm^9^, ^10^, ^11^ based on the initial work of McLean and von Fraunhofer that examined 1000 restorative gaps over a 5‐year period.^12^

Previous studies comparing milling units from different manufacturing generations have shown that bur diameter size,^13^, ^14^ the milling modes used^15^ and the number of milling axes^14^, ^16^, ^17^ have significant impacts on the accuracy of fit of milled crowns. The evidence on the accuracy of repeatedly milling crowns is limited and the effects of repetitive milling on crown surface roughness have yielded no common consensus^18^, ^19^ despite observation of bur degradation over time.^20^, ^21^ Studies exploring the marginal gaps of sequentially milled crowns have been performed on glass ceramic, zirconia and titanium prostheses only.^22^, ^23^ There are no known studies that have compared the marginal gaps of sequentially milled LDS crowns using different milling units, and specifically comparing a chairside and laboratory milling unit.

The purpose of this study was to compare the mean marginal gap (MMG) of sequentially milled CAD/CAM LDS crowns using two different milling units. The null hypotheses were:

(1) There was no difference in the overall MMG of LDS crowns constructed using the two milling units.
(2) There was no difference in the individual location MMG of LDS crowns constructed when analysed by separate milling unit and by comparison between the milling units.
(3) There was no difference in the MMG of sequentially constructed LDS crowns for each milling unit.

## MATERIALS AND METHODS

Ethics approval was not required by the relevant human research ethics committee.

### Crown designs

In a previous study,^24^ 24 Columbia typodont model (Columbia Dentoform, Long Island City, NY, USA) lower left first molars were prepared for full‐coverage LDS (IPS e.max, Ivoclar Vivadent) crowns by fourth year undergraduate students in a simulation clinic. In a subsequent study,^25^ the preparations were scanned with a TRIOS 3 scanner (3Shape, Copenhagen, Denmark) and LDS crowns designed using a Sirona inLab CAD Software Digital Design (Dentsply Sirona, Bensheim, Germany) then milled using a Sirona inLab MC X5 mill (Dentsply Sirona, Bensheim, Germany). In this study, a selection criterion was applied to rank the original crown preparations for reduction, taper, surface preparation smoothness and smoothness of margins. The crown preparation with the highest overall rank was selected for this study.

### Crown fabrication

The crown design for the selected crown preparation was obtained from the previous study.^25^ The Standard Tessellation Language (STL) file was sourced and imported into the E4D mill (E4D Dentist, D4D Technologies, Richardson, TX, USA) (E4DM) and the Sirona inLab MC X5 mill (MCX5) for milling of the LDS crowns. IPS e.max CAD (C14 A2 LT) LDS blocks were used (Ivoclar Vivadent) and new burs were inserted into the E4DM and MCX5 units prior to the commencement of the milling process. The same crown order was sent to each mill 12 times to construct a total of twelve crowns for each milling unit. Crowns were labelled E1–E12 for the E4DM and MC1‐MC12 for the MCX5. Twenty‐four crowns were produced by the two different milling units. A new set of burs was inserted when the milling unit indicated one or more burs was not able to be used further. Following milling, the crowns were desprued and crystallised in a furnace (Programat P710, Ivoclar Vivadent). The LDS crowns were not glazed, stained or adjusted in any way before or after firing.

### Marginal gap measurement

**Fig. 1:**
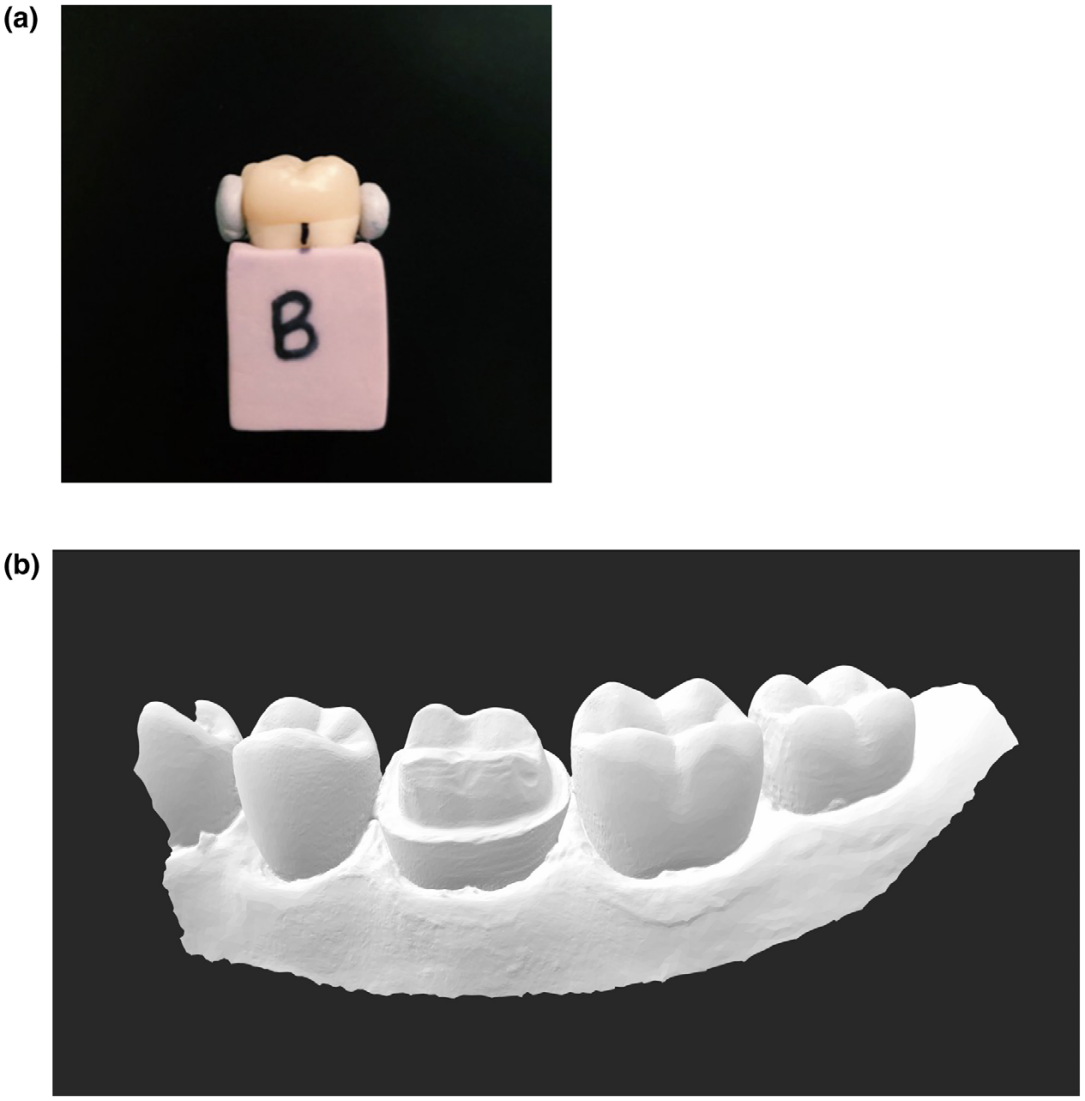
(a) Lithium disilicate crown seated onto its corresponding original crown preparation secured in a polyvinylsiloxane base. (b) Scanned crown preparation. [Colour figure can be viewed at wileyonlinelibrary.com]

**Fig. 2:**
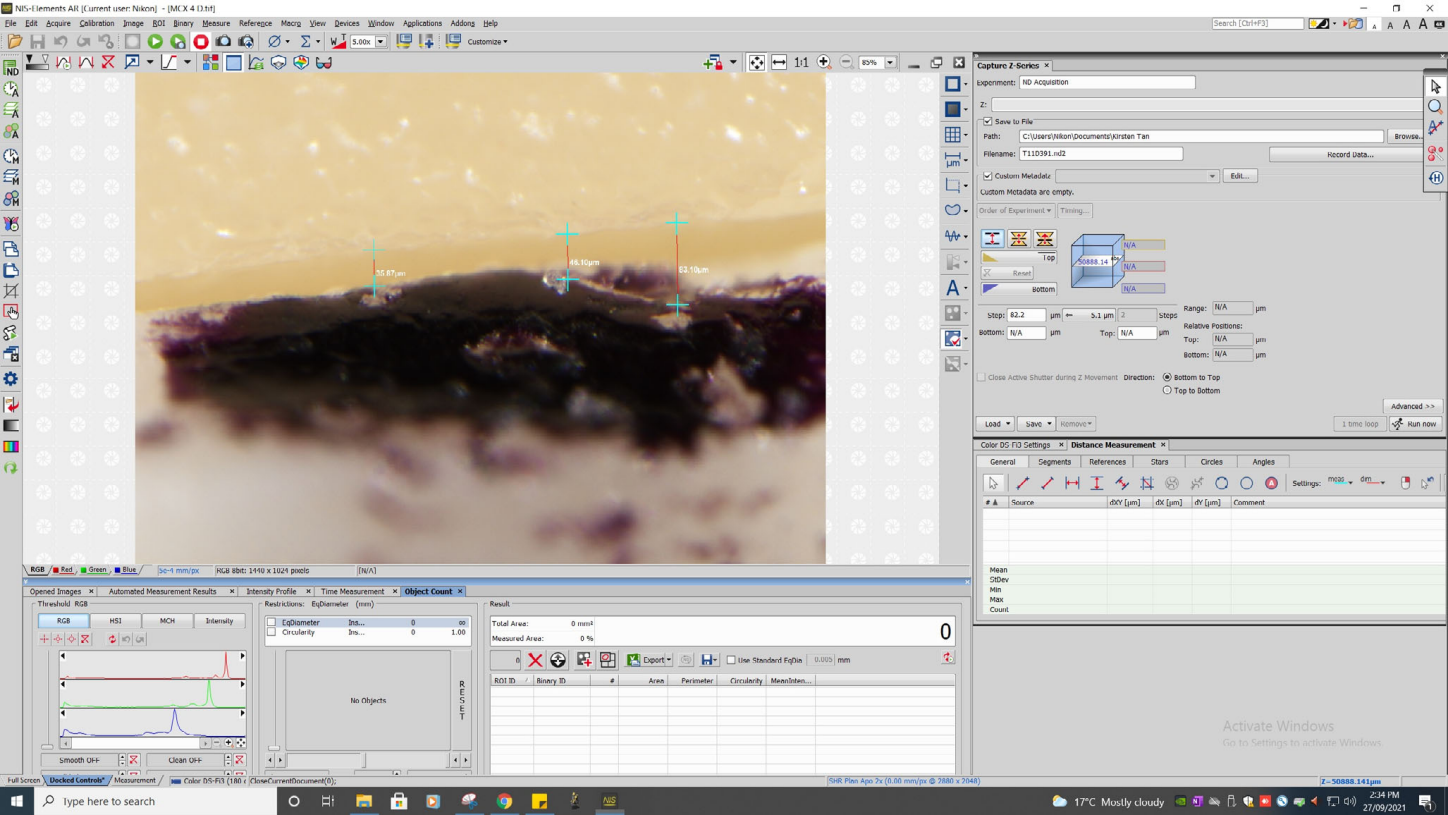
Example of the marginal gap measurement at three points per surface using the Nikon NIS‐Elements AR‐Duo control software. [Colour figure can be viewed at wileyonlinelibrary.com]

**Fig. 3:**
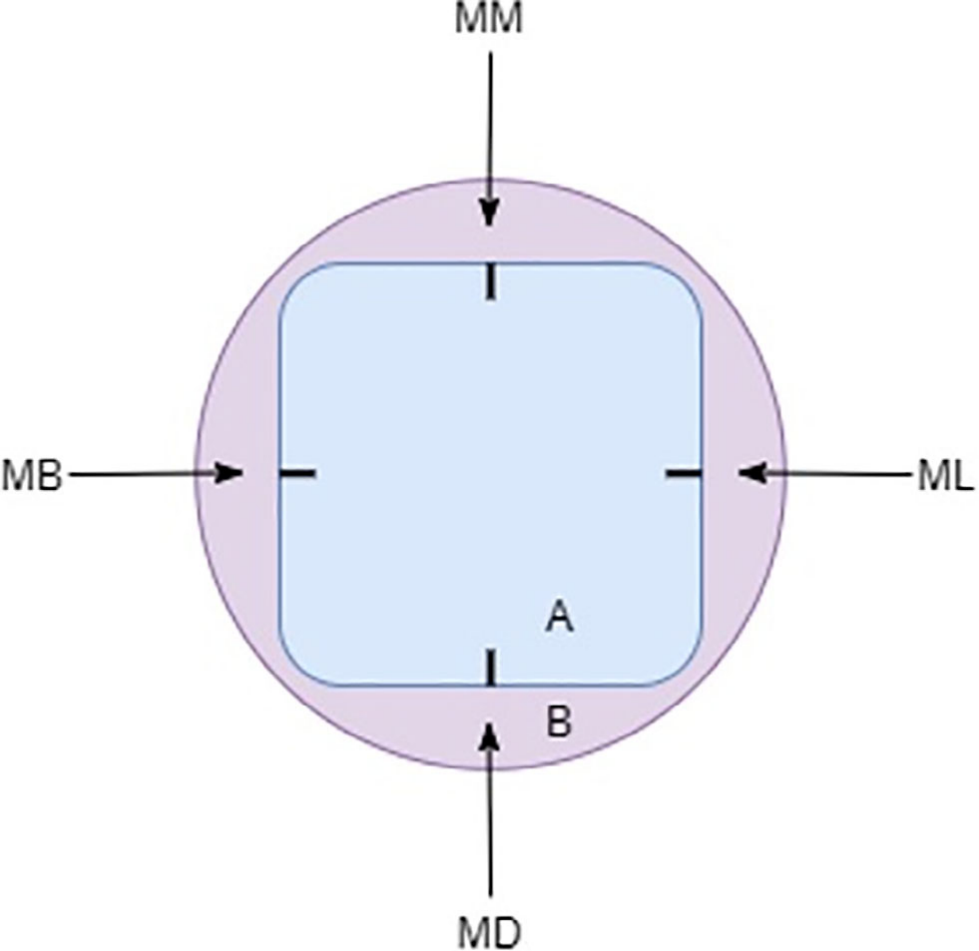
Marginal gap measurement locations (A = seated crown; B = polyvinylsiloxane base; MB = mid‐buccal; ML = mid‐lingual; MM = mid‐mesial; MD = mid‐distal). [Colour figure can be viewed at wileyonlinelibrary.com]

Each LDS crown was seated onto the original crown preparation with firm digital pressure and secured with a removable adhesive in a polyvinylsiloxane base (Fig. 1 and 1b) with four indicators to standardise the measurement locations (mid‐buccal, mid‐distal, mid‐lingual and mid‐mesial). The visco‐elastic removable adhesive adapted over the contours of the crown and tooth and permitted temporary fixation of the crown to the crown preparation without damaging the samples. The custom‐made polyvinylsiloxane base allowed the tooth to lie flat in a standardised position. The seated crowns were positioned perpendicular to the stereomicroscope (Nikon SMZ25, Nikon Instruments, Melville, NY, USA). An extended depth of focus tool was used to account for variations in the z‐plane caused by horizontal discrepancy and allowed measurement of the true vertical marginal discrepancy. A 2.0× objective lens at a fixed magnification range of 8.0–12.0× and zoom range of 4.0–9.0× was used. A DS‐Ri1 CCD camera (Nikon Instruments) mounted on the microscope transmitted live images to the Nikon NIS‐Elements AR‐Duo control software (Nikon Instruments) to measure the vertical distance between the external surface of the crown margin and the preparation finish line (Fig. 2). Three measurements were taken at each of the four sites for each crown resulting in 12 measurements per tooth and 144 measurements per milling unit (Fig. 3). All measurements were taken by the same operator.

### Statistical analysis

All measurements for all tooth surfaces were averaged to calculate an overall MMG for the E4DM and MCX5 units. The MMG for each crown was calculated by averaging all measurements for all individual locations. For each milling unit, the MMG for each location (mid‐buccal, mid‐distal, mid‐lingual, mid‐mesial) was calculated by averaging all measurements for the individual location. A linear mixed‐effects model was used for regression modelling and an F‐test for comparison of the variances. A Jonckheere‐Terpstra test was used to analyse the trend of MMG’s over time (SAS 9.4, SAS Institute, Cary, NC, USA). The level of statistical significance was set at *P* = 0.05. The threshold for being considered clinically acceptable was set at 120 μm.^12^

### Bur wear

The burs from each milling unit were viewed under a scanning electron microscope (Fei ESEM Quanta 450, FELMI‐ZFE, Graz, Austria) at 500× magnification and imaged using microscope control software (xT, FEI, Hillsboro, OR, USA) before milling commenced, at a mid‐milling sequence point and when the bur was indicated for replacement. Bur surface topography was captured using secondary electron imaging and surface composition was captured using backscatter imaging.^26^ There was no established and standardised method available to quantitatively measure the wear and diamond particle loss so this was limited to observation.

## RESULTS

### Overall MMG

The MMG for the E4DM (100.40 ± 11.69 μm) was not significantly different to the E4DM (101.08 ± 11.25 μm) unit (*P* = 0.8809). A comparison of the standard deviation between the two scanners revealed no significant difference in variability (*F* = 1.03, *P* = 0.9183).

### Individual location MMG

**Fig. 4:**
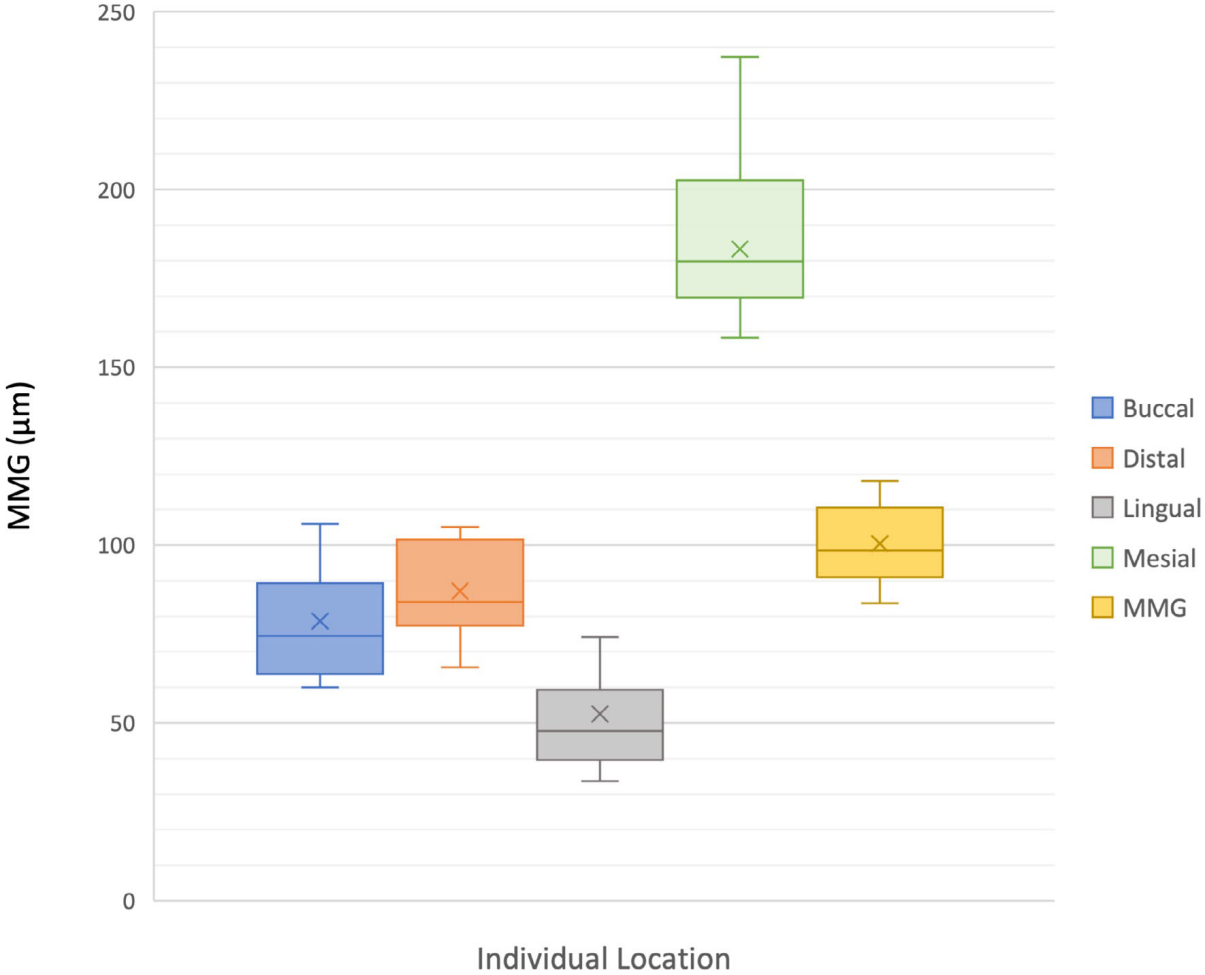
MMG for E4DM by individual location. [Colour figure can be viewed at wileyonlinelibrary.com]

**Fig. 5:**
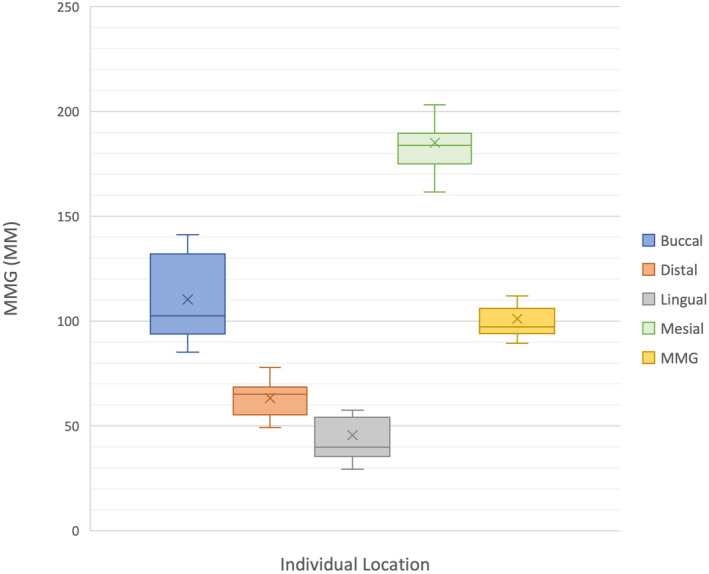
MMG for MCX5 by individual location. [Colour figure can be viewed at wileyonlinelibrary.com]

Within the E4DM and MCX5 groups, the linear mixed‐effects model revealed a significant difference in MMG between locations (*P* < 0.0001). In both the E4DM and MCX5 groups, the mid‐mesial margin recorded the largest MMG and was above the clinically accepted threshold of 120 μm (Figs 4 and 5), whereas the mid‐lingual margin recorded the lowest MMG.

In comparing the two milling units, the MMG of the mid‐buccal and mid‐distal surfaces were the two surfaces that showed a statistically significant difference from each other (*P* < 0.001).

### MMG with sequentially milled crowns

**Fig. 6:**
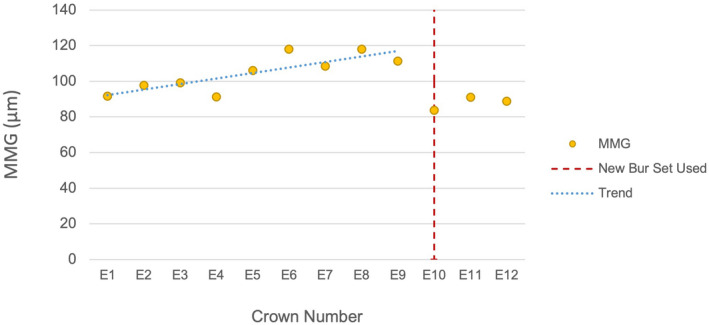
MMG of E4DM with sequentially milled crowns using the same burs. [Colour figure can be viewed at wileyonlinelibrary.com]

**Fig. 7:**
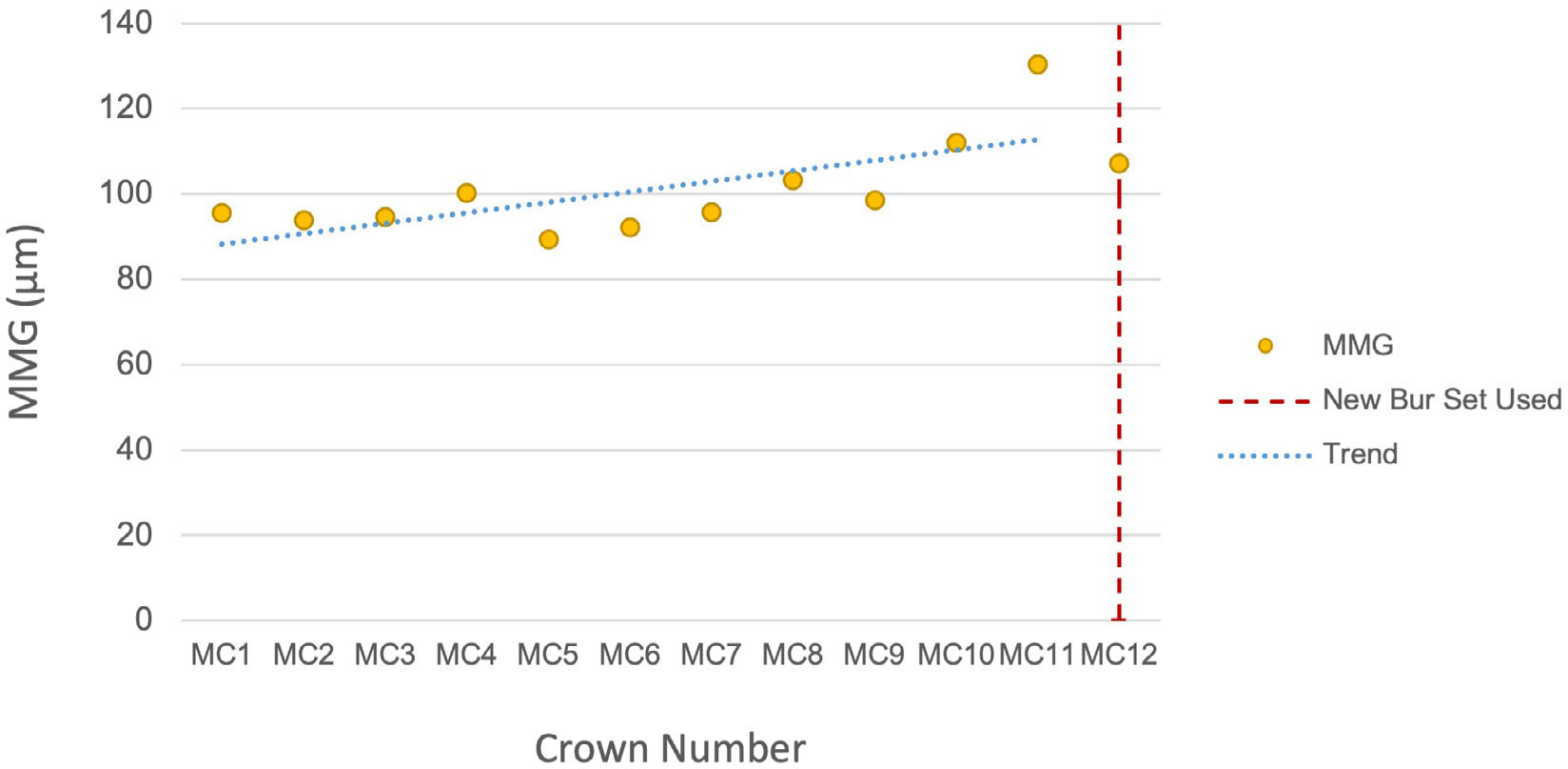
MMG of MCX5 with sequentially milled crowns using the same burs. [Colour figure can be viewed at wileyonlinelibrary.com]

The Jonckheere‐Terpstra Test revealed a significant trend of increasing MMG with sequentially milled crowns using the same burs in the E4DM (*P* = 0.0133) and MCX5 (*P* = 0.0240) groups (Figs 6 and 7). Following the replacement of the burs, the MMG reduced to a lower value that approximated that of the initial crowns milled in the sequence. The MMG for each of the 24 crowns constructed was below the clinically accepted threshold except for MC11 which was the final crown milled before the MCX5 burs required changing.

### Bur wear with sequentially milled crowns

**Fig. 8:**
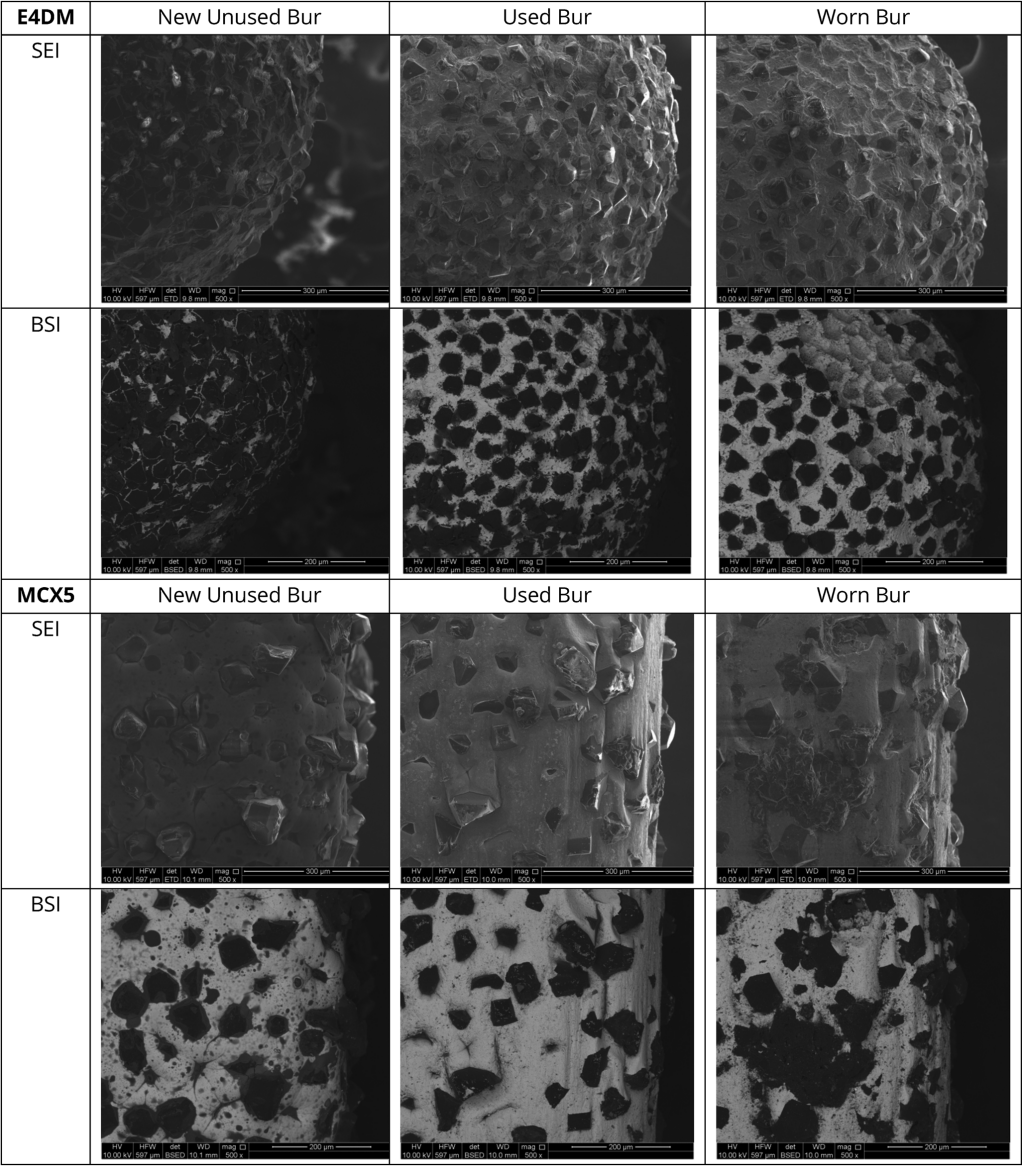
Bur wear was observed with sequential milling at 500× magnification using the scanning electron microscope (SEI = secondary electron imaging; BSI = backscatter imaging) E4DM: New (6/6 remaining), used (4/6 remaining, after E4), worn (1/6 remaining, after E9) MCX5: New (100% remaining), used (64% remaining, after MC4), worn (0% remaining, after MC11).

The images of bur wear with sequential milling are presented in Fig. 8. The blunting of the burs was evidenced by loss of the nickel matrix holding the diamond particles as well as loss of diamond particles. The main E4DM bur displayed a more noticeable pull‐out of diamond grit particles at the bur tip while the MCX5 bur predominantly displayed abrasive loss of the nickel matrix holding the diamond particles.

## DISCUSSION

This study found no difference in the overall MMG of LDS crowns constructed using the two milling units, therefore the first null hypothesis was accepted. There was a significant difference in the individual location MMG of LDS crowns constructed by both milling units when analysed separately and also when the individual locations for the milling units were compared, and there was a significant difference in the MMG of sequentially milled LDS crowns using the same burs for each milling unit, therefore the second and third null hypotheses were rejected.

### Variability of MMG

There was great variation between the smallest and largest MMG within each milling unit. The crown preparation was recruited from a series of preparations completed by undergraduate students^24^ and contained common pitfalls such as uneven preparation finish lines and overtapering.^11^, ^25^ Interproximal margins are a challenging area to prepare in inexperienced hands and the mesial margin was conceivably the more challenging.^25^ Previous analyses of the crown preparations in this study series revealed that preparations were prone to uneven or jagged finish lines in the mesial and distal regions.^27^ It was foreseeable that students had difficulty viewing and accessing the mesial margin as typically indirect vision was required. The accurate scanning of sub‐optimally refined margins could have resulted in reduced accuracy of fit which aligns with previous reasoning that preparation errors rather than milling had a significant effect on marginal gap.^11^, ^16^, ^25^, ^28^ Additionally, poor surface finish of the preparation might cause premature binding and prevent complete seating of the crown on the tooth, resulting in an artificially enlarged marginal gap.^11^

### Individual location MMG

The E4DM and MCX5 groups displayed significantly different individual location MMG’s for both mid‐buccal and mid‐distal surfaces (Figs 4 and 5). This is proposed to be attributable to STL file conversion and/or data interpretation differences between the older E4DM unit and the newer MCX5 unit. However, it does not explain the similar mid‐lingual and mid‐mesial margins which were the most accurate and least accurate, respectively, in both groups. It is possible that both milling units were less discerning for the mid‐lingual margin as there is a limit to the additional accuracy possible as the marginal gap more closely approaches the ideal value of 0 μm. A larger sample size and greater number of measurements per crown would assist in corroborating this finding.

### Increasing MMG with sequentially milled crowns

The trend of increasing MMG with sequentially milled crowns using the same burs was observed in both milling groups and might be attributable to diamond bur wear leading to poorer cutting efficacy. Changes to the working end surface of a diamond bur such as wearing down and loss of diamond particles, loss of matrix and the introduction of defects into the diamond particle microstructure affect the accuracy of the milling process by diminishing cutting efficacy which might in turn negatively impact the marginal fit.^29^ A decrease in cutting ability might lead to increased friction and stress on the ceramic block which might translate to defects and microfractures on the ceramic leading to larger marginal gaps.^29^ It has been reported that increased use of the diamond bur resulted in greater gap readings on the intaglio of milled ceramic crowns suggesting that loss of abrasive particles resulting in bur wear might be linked with a poorer crown to tooth adaptation.^30^

### Variability in bur wear rates between milling units

The MCX5 constructed 11 LDS crowns before the unit requested a change of the main milling bur, whereas the E4DM requested a bur change after nine crowns (Figs 6 and 7). Both milling units produced LDS crowns with an overall MMG within a clinically acceptable range with the exception of MCX5 crown MC11 which was the last crown constructed before the mill requested a bur change. While the E4DM used a single dedicated 1.1 mm rounded tip bur for milling the internal surface, the MCX5 used four burs of diameters of 2.2, 1.4, 1.2 and 0.6 mm interchangeably to progressively mill the intaglio and external crown surfaces.^25^ There was a more even distribution of bur wear and loss of diamond particles across the four burs, as observed in the scanning electron microscope images. The greater number of burs used and differential rate of bur wear explains the MCX5 unit milling a larger number of crowns before the burs required changing in comparison to the E4DM unit. The recognition and measurement of bur wear are challenging and relies either on the unit to detect bur wear or on applying a limit to the number of crowns milled with a bur set before replacement.

### Differences in milling units

The E4DM was released to the marketplace approximately 10 years ago and is a 3‐axis chairside mill whereas the MCX5 unit is a more recent 5‐axis commercial mill. While there have been great advances in milling technology and efficiency, the results of the current study suggest the two milling units had no significant influence on the marginal gap of LDS crowns. Instead, the proposed technological advances might have had a greater impact on milling efficiency, ease of operator use and advertising potential.

### Limitations

The crown preparation selected for this study was performed by one of a group of undergraduate students on a manikin in a simulation clinic environment which allowed reproduction of some but not all clinical factors. On one hand, the crown preparation was not an ideal or benchtop preparation and on the other hand, it did not include potentially confounding factors such as blood, saliva, access and patient compliance that are typically present *in vivo*.^31^, ^32^, ^33^ The crown preparation was limited to a lower left molar and it is acknowledged typodont teeth have different to natural tooth structure in surface hardness.^34^ Student crown preparations are prone to overtapering which might make the crown more susceptible to ‘rocking’ on the preparation giving rise to artificially enlarged and diminished marginal gaps on one side and its contralateral side.^27^ An ideal preparation by an experienced operator might yield different research outcomes.^11^

The marginal gap involves three dimensions and this study acknowledges the external vertical marginal gap was measured at selected locations and extrapolated to represent the overall crown marginal gap.^9^ Three‐dimensional measurement methods such as microcomputed tomography would enhance the accuracy and reliability and facilitate further investigation of the effect of bur wear on crown fit by assessing both the internal fit and horizontal discrepancy.^4^, ^13^, ^35^, ^36^ It would be ideal to obtain measurements at a large number of different locations using a large sample size. In the current study, the measurement protocol was standardised across all samples and performed by a single trained researcher. Future research might investigate a wider array of milling units and ceramic materials and develop a quantitative measurement of bur wear to examine its effect on the quality of the milled crown.

Within the limitations of this *in vitro* study, the E4DM and MCX5 milling units produced LDS crowns with similar overall MMG’s and within a clinically acceptable range. There was a statistically significant difference in the individual location MMG of LDS crowns constructed by both milling units when analysed separately and also when the individual locations for the milling units were compared. For both milling units, there was a statistically significant trend of increasing MMG with sequentially milled crowns using the same burs.

## Disclosure Statement

There is no conflict of interest to declare.

## Funding

The authors would like to thank the Australian Prosthodontic Society for their support through the Australian Prosthodontic Society Research Grant.

## Ethics

The study was performed at The University of Adelaide and ethics approval was not required by The University of Adelaide Human Research Ethics Committee.
